# *MIR‐138‐5P* inhibits the progression of prostate cancer by targeting *FOXC1*

**DOI:** 10.1002/mgg3.1193

**Published:** 2020-02-28

**Authors:** Hui Huang, Ying Xiong, Zhensheng Wu, Yuhui He, Xianglin Gao, Zhangyan Zhou, Tao Wang

**Affiliations:** ^1^ Department of Urology The Affiliated Shunde Hospital of Guangzhou Medical University Foshan China; ^2^ Department of Urology Yangtze University, Affiliated Hospital 1 Jingzhou China

**Keywords:** *FOXC1*, *MIR‐138‐5P*, prostate cancer, tumor suppressor gene

## Abstract

**Background:**

Studies have suggested that micro‐RNAs (miRNAs) can function as an oncogene or a tumor suppressor in cancers. However, the role of *MIR‐138‐5P* (613394) in prostate cancer (PCa) remains unclear.

**Methods:**

Expression level of *MIR‐138‐5P* in PCa cell lines and normal cell line was analyzed with the quantitative real‐time PCR method. Cell counting kit‐8 assay, colony formation assay, wound‐healing assay, and transwell invasion assay were performed to analyze the biological functions of *MIR‐138‐5P*.

**Results:**

We showed *MIR‐138‐5P* expression level was significantly decreased in PCa cell lines compared with the normal cell line. Overexpression of *MIR‐138‐5P* inhibits PCa cell proliferation, colony formation, cell migration, and cell invasion in vitro. Mechanistically, we showed Forkhead box C1 (*FOXC1*, 601090) was a direct target for *MIR‐138‐5P* in PCa. We confirmed that overexpression of FOXC1 partially reversed the effects of *MIR‐138‐5P* on PCa cell behaviors.

**Conclusions:**

Collectively, we showed that *MIR‐138‐5P* functions as a tumor suppressor gene in PCa via targeting *FOXC1*.

## Graphical abstract

FOXC1 overexpression could increase PCa cell proliferation, colony formation, migration, and invasion. Overexpression of FOXC1 could attenuated the effects of miR‐138‐5p on PCa cell behaviors.

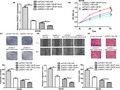

## 1 INTRODUCTION

Prostate cancer (PCa) is a big health threat for men worldwide with increasing incidence (Castillejos‐Molina & Gabilondo‐Navarro, 2016). What is worse, the pathogenesis of PCa remains largely unknown, which results in the lack of treatment options, leading to worse overall survival of PCa patients (Murillo‐Garzón & Kypta, 2017). Hence, further investigations are needed to understand the mechanisms behind PCa progression.

Noncoding RNAs (ncRNAs) including microRNA (miRNA), long noncoding RNA (lncRNA), and circular RNA (circRNA) have been revealed to function as crucial roles in PCa progression (Greene et al., 2019; Kanwal, Plaga, Liu, Shukla, & Gupta, 2017; Wu, Xiao, Zhou, Zhou, & Yan, 2019). Increasing evidence suggested that ncRNAs can be developed as biomarkers for prognosis prediction and treatment (Greene et al., 2019; Kanwal et al., 2017; Wu et al., 2019). miRNAs are short RNAs (18–24 nucleotides in length) without protein‐coding capability (Ha & Kim, 2014). Dual functions of miRNAs in cancers have been reported, as some miRNA can promote cancer development, while some of them can inhibit tumorigenesis (Suzuki, Maruyama, Yamamoto, & Kai, 2013). By 3′‐untranlated region (3′‐UTR) binding, miRNAs can regulate multiple gene expressions, associated signaling pathways, and eventually affect the hallmarks of cancer (Acunzo, Romano, Wernicke, & Croce, 2015).

*MIR‐138‐5P* (613394, NC_000003.12) is an miRNA that reported to function as a tumor suppressive role in several human cancers. For instance, *MIR‐138‐5P* was revealed to be a decreased expression in colorectal cancer, and its low expression was significantly correlated with advanced tumor stage and poor overall survival (Zhao et al., 2016). Additionally, programmed cell death ligand 1 was identified as a direct target for *MIR‐138‐5P*, indicating *MIR‐138‐5P* has the potential to regulate immune response (Zhao et al., 2016). Besides that, *MIR‐138‐5P* was also identified to be a reduced expression in pancreatic cancer and suppressed autophagy and tumor growth through regulating the *SIRTUIN 1*(604479)*/forkhead box O1* (136533)*/RAS‐ASSOCIATED PROTEIN RAB7* (602298) axis (Tian, Guo, Yu, Sun, & Jiang, 2017). In non‐small‐cell lung, *MIR‐138‐5P* was found could affect the response of cancer cell to cisplatin through regulating *ATG7* (608760; Pan, Chen, Shen, & Tantai, 2019). Moreover, lncRNA *RP11‐476D10.1* (600138) was revealed and could function as a sponge for *MIR‐138‐5P* to regulate *leucine‐rich repeat kinase 2* (609007) expression in papillary thyroid carcinoma (Zhao, Zhao, Li, & Zhong, 2019). Overexpression of *MIR‐138‐5P* was shown to promote apoptosis and autophagy of papillary thyroid carcinoma cells (Zhao, Zhao, et al., 2019). There is a previous work to indicate *MIR‐138* could modulate PCa migration and invasion but did not indicate whether it was the 5p or the 3p strand (Yu, Wang, Li, Yang, & Tang, 2018). However, the biological roles of *MIR‐138‐5P* in PCa remain to be explored.

*Forkhead box C1* (*FOXC1*, 601090, NG_009368.1), located at chromosome 6p25, is a transcription factor that belongs to the FOX gene family (Nishimura et al., 1998). *FOXC1* has found to not only have crucial roles in normal physiological and pathological conditions but also shown to be important mediators for cancer progression (Han et al., 2017). For example, the knockdown of *FOXC1* was found to inhibit glioma cell epithelial‑to‑mesenchymal transition via regulating the β‐catenin signaling (Cao et al., 2019).

In this work, we explored the expression level of *MIR‐138‐5P* in PCa cell lines. Furthermore, gain‐of‐function experiments were performed to investigate the biological roles of *MIR‐138‐5P* in PCa. Moreover, we predicated the targets of *MIR‐138‐5P* at TargetScan and *FOXC* was selected for the following analyses.

## 2 MATERIALS AND METHODS

### 2.1 Cell culture

PCa cell lines (PC3 and DU145) were seeded into F‐12K medium in supplement with 10% fetal bovine serum (FBS) purchased from Thermo Fisher Scientific, Inc. Normal prostate epithelial cells (RWPE‐1) were cultured in Dulbecco's modified Eagle's medium supplemented with 10% FBS obtained from Thermo Fisher Scientific, Inc. The incubation atmosphere was maintained at 37°C containing 5% of CO_2_. All these cell lines used were purchased from Cell Bank of Chinese Academy of Sciences.

### 2.2 Cell treatment

*MIR‐138‐5P*mimic (5′‐AGCUGGUGUUGUGAAUCAGGCCG‐3′) and the corresponding negative control (NC‐miR, 5′‐GCGGUCGUGCAGUGCGUGAUAUA‐3′) were synthesized by RiboBio Inc. *MIR‐138‐5P* overexpression was accompanied by transfecting *MIR‐138‐5P* mimic into PCa cell lines using Lipofectamine 2000 (Invitrogen, Thermo Fisher Scientific, Inc.). pcDNA3.1 contains the coding sequence of *FOXC1* (pcFOXC1) and an empty vector were purchased from GenScript. Transfection was also conducted using Lipofectamine 2000 using the manufacturer's instruction.

### 2.3 Quantitative real‐time PCR (RT‐qPCR)

RNA from the cells was isolated with TRIZOL reagent (Beyotime). After concentration determination, RNA was reverse transcribed into complementary DNA with PrimeScript RT Reagent (Takara). RT‐qPCR was conducted at ABI 7500 system (Applied Biosystems) using SYBR Green Mix (Takara). The method of 2 − ΔΔCt was used to measure the relative expression level of *MIR‐138‐5P* or *FOXC1* using *U6 small nuclear RNA* (U6 snRNA, 180692) or *glyceraldehyde‐3‐phosphate dehydrogenase* (*GAPDH*, 138400) as endogenous control. Primers used were as follows: *MIR‐138‐5P*: Forward: 5′‐GCGAGCTGGTGTTGTGAATC‐3′, Reverse: 5′‐AGTGCAGGGTCCGAGGTATT‐3′; *U6 snRNA*: Forward: 5′‐CTCGCTTCGGCAGCACA‐3′, Reverse: 5′‐AACGCTTCACGAATTTGCGT‐3′; *FOXC1*: Forward: 5′‐CGGTATCCAGCCAGTCTCTGTACG‐3′, Reverse: 5′‐GTTCGGCTTTGAGGGTGTGTC‐3′; *GAPDH*: Forward: 5′‐GGAGGGAGATCCCTCCAAAAT‐3′, Reverse: 5′‐GGCTGTTGTCATACTTCTCATGG‐3′. Experiments were repeated in triplicate.

### 2.4 Cell counting kit‐8 (CCK‐8) assay

Cells in the density of 1 × 10^4^ cells/well were seeded into 96‐well plates. After 0, 24, 48, or 72 hr incubation, CCK‐8 reagent purchased from Beyotime was filled into the plate and further incubated for 4 hr. Optical density in each well was analyzed at a wavelength of 450 nm. Experiments were repeated in triplicate.

### 2.5 Colony formation assay

Cells were incubated into 6‐well plate at the density of 800 cells/well. Colonies generated from the cultured cells were fixed with methanol, stained with crystal violet, and then counted under the microscope. Experiments were repeated in triplicate.

### 2.6 Cell migration assay

Cells were seeded in 6‐well plates and incubated until about 80% confluence. The pipette tip was used to create a wound at the cell surface. Thereafter, the cells were washed with PBS hree times to remove cell debris. After incubation for 0 or 48 hr, cell images were captured under the microscope to evaluate the effects of *MIR‐138‐5P* or *FOXC1* expression on cell migration. Experiments were repeated in triplicate.

### 2.7 Cell invasion assay

In this experiment, 1 × 10^5^ cells in serum‐free medium were filled into the upper chamber of the Matrigel‐coated insert. The lower chamber was filled with medium contains FBS. After incubation for 48 hr, noninvasive cells were removed with cotton. Then, invasive cells were fixed with methanol, stained using crystal violet, and counted under microscope. Experiments were repeated in triplicate.

### 2.8 Bioinformatic analysis

TargetScan was used to detect the putative targets for *MIR‐138‐5P*. Among all these targets, *FOXC1* was selected for the following analyses.

### 2.9 Luciferase activity reporter assay

To validate the direct connection of *MIR‐138‐5P* and *FOXC1*, luciferase activity reporter assay was performed. The wild‐type (wt) or mutant (mt) 3′‐UTR sequence of *FOXC1* was inserted into psiCHECK‐2 to obtain wt‐FOXC1 or mt‐FOXC1 luciferase vectors. For the luciferase activity reporter assay, cells were co‐transfected with luciferase vectors and miRNAs using Lipofectamine 2000. After transfection for 48 hr, relative luciferase activity was measured with a dual‐luciferase reporter kit (Promega) with Renilla luciferase activity as the internal control. Experiments were repeated in triplicate.

### 2.10 Statistical analyses

Data were obtained from three independent experiments and then expressed as mean ± *SD* after analyses using GraphPad Prism 6.0 (GraphPad Software). Differences in groups were assessed with Student's *t* test or one‐way ANOVA and Tukey post hoc test. *p* value less than .05 was considered as a statistically significant difference.

## 3 RESULTS

### 3.1 Decreased expression of *MIR‐138‐5P* in PCa cells

**Figure 1:**
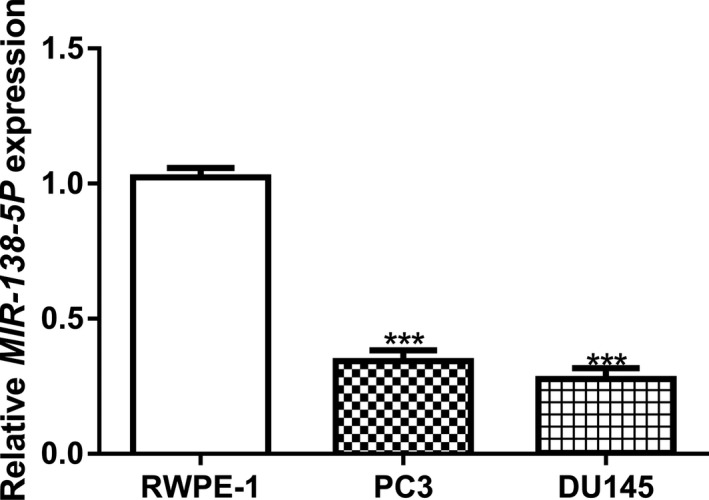
*MIR‐138‐5P* expression was decreased in PCa cells compared with normal cell line. *MIR‐138‐5P*: *MICRORNA‐138‐5P* (***p < .001). PCa, prostate cancer

To explore the function of *MIR‐138‐5P*, we first analyzed its expression level in PCa cell lines and in the normal cell line. We found the *MIR‐138‐5P* expression level was significantly decreased in PCa cells compared with the normal cell line (Figure 1).

### 3.2 *MIR‐138‐5P* overexpression inhibits PCa cell growth, migration, and invasion

**Figure 2:**
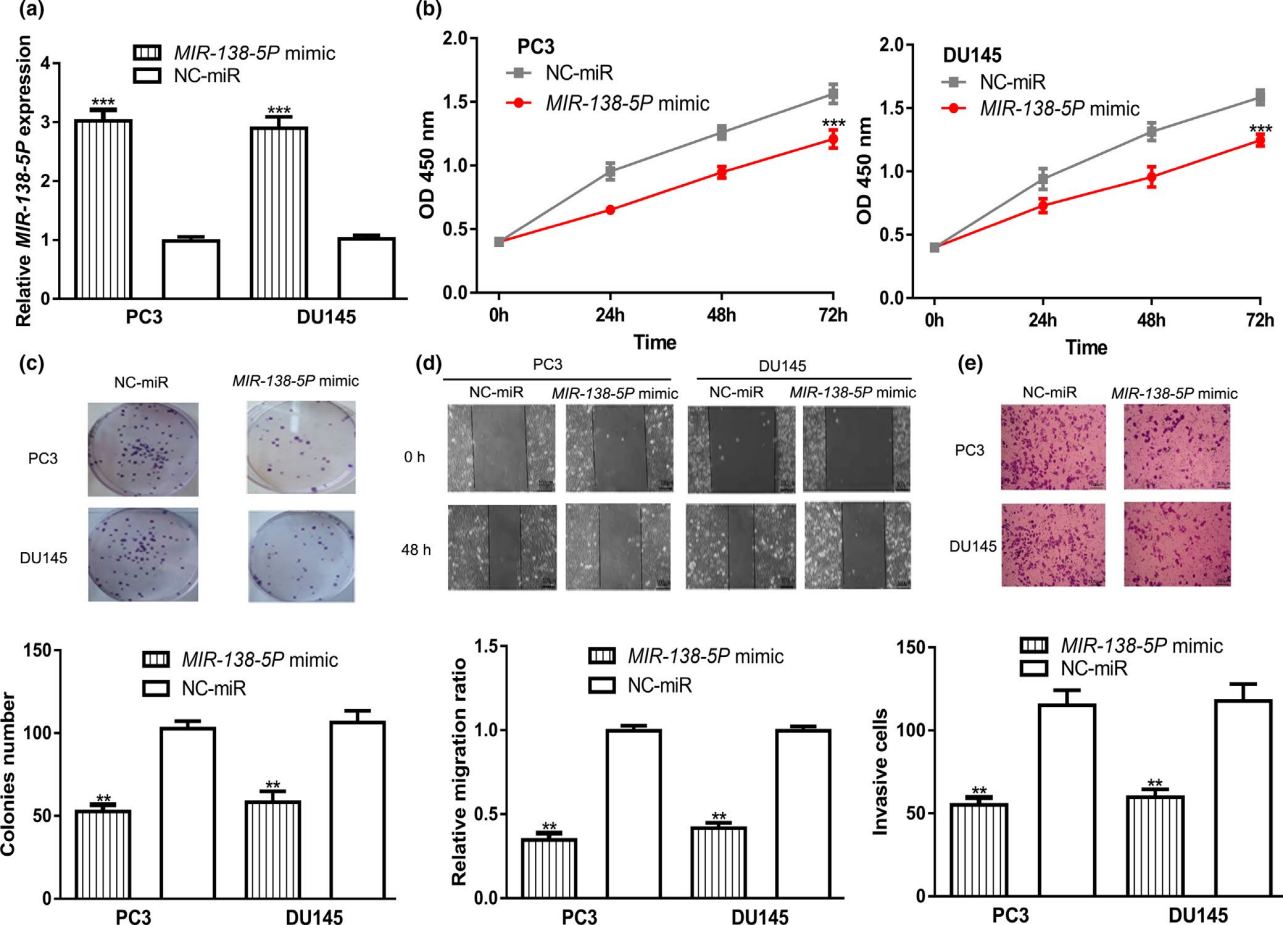
Transfection of *MIR‐138‐5P* mimic inhibited PCa cell proliferation, colony formation, cell migration, and cell invasion. (a) *MIR‐138‐5P* expression, (b) Cell proliferation, (c) Colony formation, (d) Cell migration, and (e) Cell invasion in PCa cells transfected with *MIR‐138‐5P* mimic or NC‐miR (**p < .01, ***p < .001). *MIR‐138‐5P*, *MICRORNA‐138‐5P*; NC‐miR, negative control microRNA; PCa, prostate cancer

We then detected the roles of *MIR‐138‐5P* overexpression on PCa cells. Introduction of *MIR‐138‐5P* mimic significantly increased *MIR‐138‐5P* levels in PCa cells (Figure 2a). CCK‐8 assay and wound‐healing assay revealed that the overexpression of *MIR‐138‐5P* inhibits PCa cell proliferation and colony formation (Figure 2b,c). Moreover, we found in PCa cells transfected with *MIR‐138‐5P* mimic cell migration and invasion abilities were significantly inhibited (Figure 2d,e).

### 3.3 *MIR‐138‐5P* can interact with *FOXC1*

**Figure 3:**
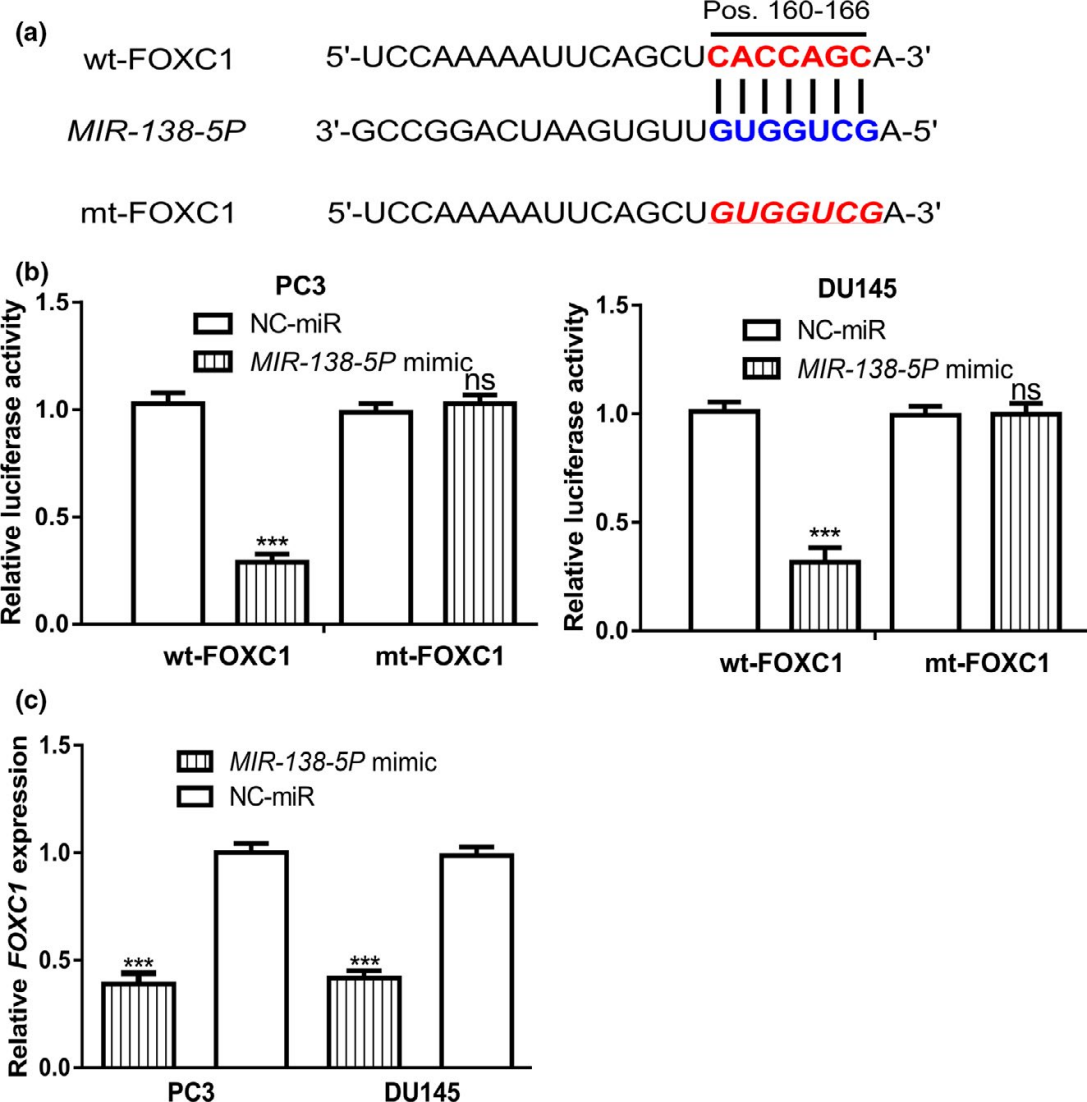
Direct interaction of *MIR‐138‐5P* and *FOXC1*. (a) Binding scheme of *MIR‐138‐5P* and the 3’‐UTR of *FOXC1*. (b) Relative luciferase activity in PCa cells transfected with wt/mt‐FOXC1 and *MIR‐138‐5P* mimic or NC‐miR. (c) Relative *FOXC1* expression level in PCa cells transfected with *MIR‐138‐5P* mimic or NC‐miR (ns, not significant; ***p < .001). *FOXC1*, Forkhead box C1; *MIR‐138‐5P*, *MICRORNA‐138‐5P*; mt, mutant; NC‐miR, negative control microRNA; PCa, prostate cancer; UTR, untranslated region; wt, wild‐type

Subsequently, we are interested to explore the targets of *MIR‐138‐5P* using TargetScan. We found *FOXC1* was a putative target for *MIR‐138‐5P* (Figure 3a). Luciferase activity reporter assay validated the direct connection of *MIR‐138‐5P* and the 3′‐UTR of *FOXC1* (Figure 3b). The RT‐qPCR analysis results indicated that overexpression of *MIR‐138‐5P* could inhibit the expression level of *FOXC1* in PCa cells (Figure 3c).

### 3.4 *MIR‐138‐5P* regulates PCa cell malignancy behaviors via targeting *FOXC1*

**Figure 4:**
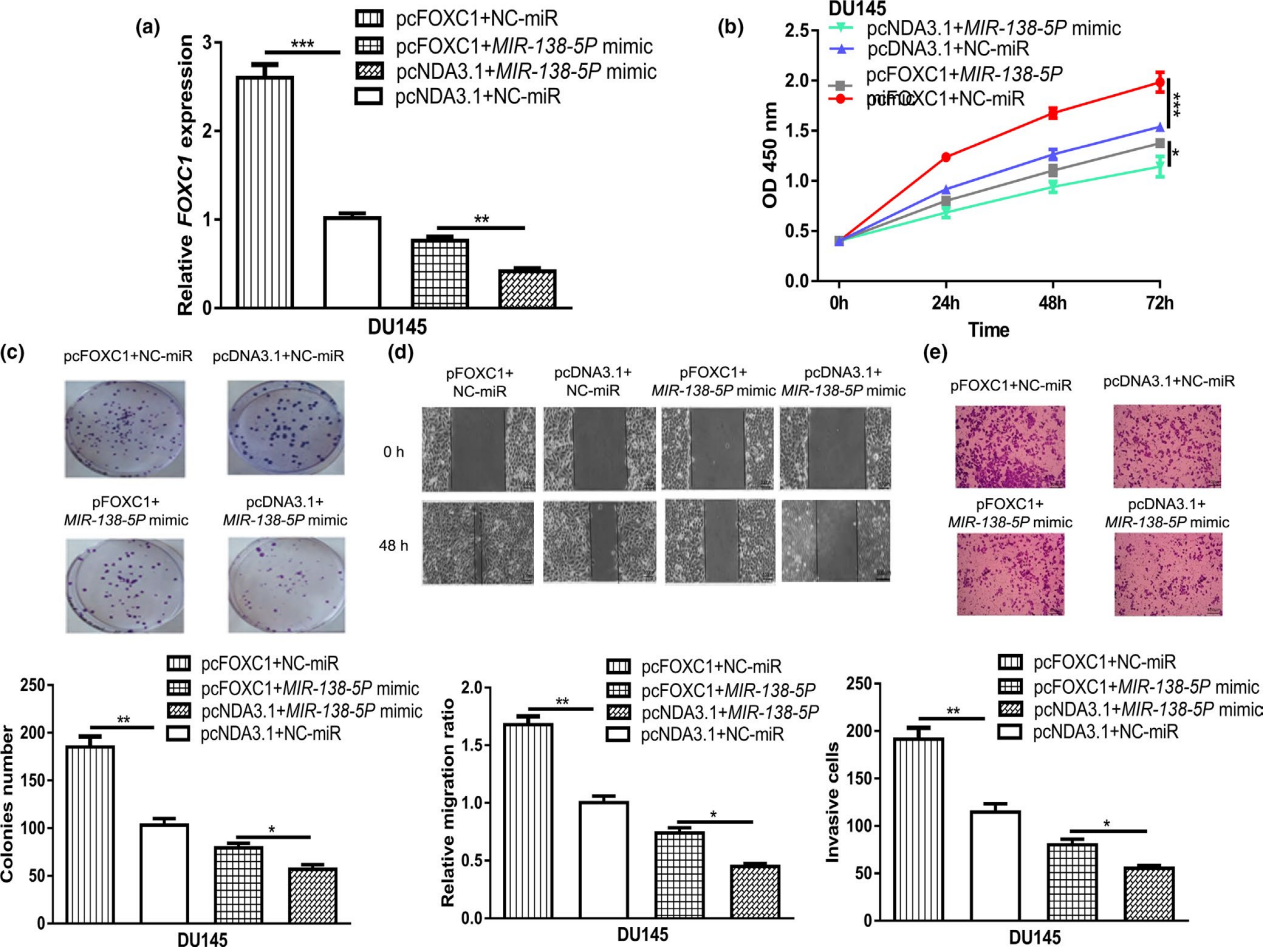
*MIR‐138‐5P* inhibits PCa progression by directly interacting with *FOXC1*. (a) *MIR‐138‐5P* expression, (b) Cell proliferation, (c) Colony formation, (d) Cell migration, and (e) Cell invasion in PCa cells transfected with pcFOXC1+NC‐miR, pcFOXC1+*MIR‐138‐5P* mimic, pcNDA3.1+NC‐miR, or pcNDA3.1+*MIR‐138‐5P* mimic (*p < .05, **p < .01, ***p < .001). *FOXC1*, Forkhead box C1; *MIR‐138‐5P*, *MICRORNA‐138‐5P*; NC‐miR: negative control microRNA; PCa, prostate cancer

To clarify the relationship between *MIR‐138‐5P* and *FOXC1*, rescue experiments were conducted. We showed pcFOXC1 transfection increased the levels of *FOXC1* in PCa cell (Figure 4a). In addition, we found the effects of *MIR‐138‐5P* mimic on *FOXC1* expression can be reversed by pcFOXC1 (Figure 4a). In vitro functional experiments showed that overexpression of *FOXC1* promoted PCa cell proliferation, colony formation, cell migration, and cell invasion (Figure 4b–e). Moreover, we showed that overexpression of *FOXC1* partially abolished the effects of *MIR‐138‐5P* mimic on PCa cell behaviors (Figure 4b–e).

## 4 DISCUSSION

miRNAs were reported to play crucial roles in the prevention or promotion of cancer progression (Acunzo et al., 2015; Suzuki et al., 2013). The miRNAs that promote carcinogenesis are the oncomiRs, while these can inhibit tumorigenesis were termed as tumor suppressive miRNAs. To date, numerous miRNAs have been identified to be aberrantly expressed in the development of PCa. For example, M*IR‐301A‐3P* (615675) was revealed to be an elevated expression in PCa tissues along with several cell lines (Fan, Wang, Huo, & Wang, 2019). In addition, they found PCa cell proliferation and invasion can be stimulated by *MIR‐301A‐3P* through regulating the expression of runt‑related transcription factor 3 (600210; Fan et al., 2019). Besides that, *MIR‐198* (605547) was revealed to be a decreased expression in both PCa tissues and cell lines (Ray et al., 2019). In addition, the tumor growth ability can be inhibited in vitro and in vivo by the *MIR‐198*/*Mindbomb E3 ubiquitin protein ligase 1* (608677) regulatory axis (Ray et al., 2019).

In this study, we showed that *MIR‐138‐5P* expression was decreased in PCa cell lines compared with the normal cell line. Previous studies demonstrated that *MIR‐138‐5P* regulates cancer migration, invasion, and epithelial–mesenchymal transition (Zhao, Ling, Li, Hou, & Zhao, 2019). Hence, we also explored the role of *MIR‐138‐5P* on cell behavior. Here we showed the restored expression of *MIR‐138‐5P* inhibits PCa cell proliferation, colony formation, cell migration, and cell invasion, indicating that *MIR‐138‐5P* functions as a tumor suppressive in PCa. Our work presented here is similar to the role of *MIR‐138‐5P* presented in other cancer types (Pan et al., 2019; Tian et al., 2017; Zhao, Zhao, et al., 2019). Several targets for *MIR‐138‐5P* have been identified in previous studies, which has helped us to understand the role of *MIR‐138‐5P* in human cancers. Hence, we also explored the potential target for*MIR‐138‐5P* in this work. Combining the results of bioinformatic analysis, luciferase activity reporter assay, and RT‐qPCR analysis, we found *FOXC1* was a putative target for *MIR‐138‐5P*. Functionally we showed that the overexpression of *FOXC1* could promote PCa cell malignancy behavior. Importantly, rescue experiments found overexpression of *FOXC1* could partially reverse the effects of *MIR‐138‐5P* on PCa cells. This work provides novel evidence regarding the mechanisms behind the progression of PCa, which could provide novel targets for cancer treatment. However, the limitation in this work was that we did not explore the function of *MIR‐139‐5P*/*FOXC1* axis in animal model, which we believe should be performed in the future.

Collectively, our work established the tumor suppressive role of *MIR‐138‐5P* in PCa. *FOXC1* was identified as the novel target for *MIR‐138‐5P*, through which *MIR‐138‐5P* exerts the inhibitory effects on PCa cells. The validated *MIR‐138‐5P* and *FOXC1* axis could help us to develop novel targets for PCa treatment.

## CONFLICT OF INTEREST

The authors declare that they have no conflict of interest.
